# ‘Intracytoplasmic sperm injection (ICSI) paradox’ and ‘andrological ignorance’: AI in the era of fourth industrial revolution to navigate the blind spots

**DOI:** 10.1186/s12958-024-01193-y

**Published:** 2024-02-13

**Authors:** Pallav Sengupta, Sulagna Dutta, Ravindran Jegasothy, Petr Slama, Chak-Lam Cho, Shubhadeep Roychoudhury

**Affiliations:** 1 Department of Biomedical Sciences, College of Medicine, Gulf Medical University (GMU), Ajman, UAE; 2 Basic Medical Sciences Department, College of Medicine, Ajman University, Ajman, UAE; 3 Faculty of Medicine, Bioscience and Nursing, MAHSA University, Kuala Lumpur, Malaysia; 4 Laboratory of Animal Immunology and Biotechnology, Department of Animal Morphology, Physiology and Genetics, Faculty of AgriSciences, Mendel University in Brno, Brno, Czech Republic; 5 S. H. Ho Urology Centre, Department of Surgery, The Chinese University of Hong Kong, Hong Kong, China; 6 Department of Life Science and Bioinformatics, Assam University, Silchar, India

**Keywords:** Andrology, Artificial intelligence, Assisted reproductive technology, Industrial revolution 4.0, Intracytoplasmic sperm injection, Male infertility

## Abstract

The quandary known as the Intracytoplasmic Sperm Injection (ICSI) paradox is found at the juncture of Assisted Reproductive Technology (ART) and ‘andrological ignorance’ – a term coined to denote the undervalued treatment and comprehension of male infertility. The prevalent use of ICSI as a solution for severe male infertility, despite its potential to propagate genetically defective sperm, consequently posing a threat to progeny health, illuminates this paradox. We posit that the meteoric rise in Industrial Revolution 4.0 (IR 4.0) and Artificial Intelligence (AI) technologies holds the potential for a transformative shift in addressing male infertility, specifically by mitigating the limitations engendered by ‘andrological ignorance.’ We advocate for the urgent need to transcend andrological ignorance, envisaging AI as a cornerstone in the precise diagnosis and treatment of the root causes of male infertility. This approach also incorporates the identification of potential genetic defects in descendants, the establishment of knowledge platforms dedicated to male reproductive health, and the optimization of therapeutic outcomes. Our hypothesis suggests that the assimilation of AI could streamline ICSI implementation, leading to an overall enhancement in the realm of male fertility treatments. However, it is essential to conduct further investigations to substantiate the efficacy of AI applications in a clinical setting. This article emphasizes the significance of harnessing AI technologies to optimize patient outcomes in the fast-paced domain of reproductive medicine, thereby fostering the well-being of upcoming generations.

## Background

In vitro fertilization (IVF), a monumental 20th century innovation, was initially developed for women with tubal disease in 1978. Its scope broadened in the 1990s with intracytoplasmic sperm injection (ICSI) to address poor semen quality and has since expanded to milder fertility issues. Although many successful IVF births have bolstered its reputation, its widened application could result in unnecessary treatments for some couples. Given emerging concerns regarding the health of IVF-conceived children, the applicability of this technique for minor fertility concerns remain debatable [1].

ICSI is a widely used technique in assisted reproductive technology (ART), where a single sperm is directly injected into an egg for fertilization [2]. It has definitely revolutionized the treatment of male infertility, particularly in cases of severe oligozoospermia or azoospermia [3]. On the contrary, the rise of technology and growing reliance of clinicians and couples on ARTs allows severe male infertility to be sidestepped through ICSI. However, this may not always be the optimal solution. As such, the conundrum of ICSI is becoming more evident, especially as it tends to overshadow the often-neglected treatment of male infertility [4], a phenomenon known as ‘andrological ignorance’. ‘Andrological ignorance’ refers to the lack of knowledge and understanding about male reproductive health, which can lead to misdiagnosis and suboptimal treatment of male infertility [5].

ICSI markedly enhances the likelihood of conception in scenarios where traditional fertilization methods are ineffective, thereby affirming its utility. As a result, ICSI emerges as a significant intervention for couples struggling with male factor infertility, providing a viable option in situations that formerly offered limited prospects. ICSI accounts for up to 80% of all ART cases. It has significantly improved the success rates of ART, especially in cases of male factor infertility, which accounts for 50% of all infertility cases [3]. Nonetheless, it is crucial to juxtapose this recognition with an awareness of the potential overutilization of ICSI in a variety of contexts. Such widespread application may inadvertently detract from a thorough investigation into the root causes of male infertility. This trend poses concerns regarding the long-term consequences of circumventing natural selection mechanisms, which could lead to the propagation of genetic anomalies. Therefore, while the role of ICSI in mitigating male infertility is substantial and commendable, its indiscriminate use necessitates prudent consideration. This is particularly relevant in light of the prevalent ‘andrological ignorance,’ which underscores the importance of a balanced and judicious application of this technique.

However, the paradox of ICSI also lies in the fact that it allows the transmission of genetically defective sperm since ICSI bypasses natural sperm selection mechanisms and introduces sperm that may not have survived the natural selection process [2]. Studies have shown that sperm selected for ICSI have a higher prevalence of genetic defects, such as chromosomal abnormalities, DNA fragmentation, and epigenetic alterations, compared to sperm selected for conventional in vitro fertilization (IVF) [6]. This raises concern about the long-term health consequences of ICSI offspring, particularly in cases of repeated ICSI cycles, which are common in severe male factor infertility cases [6]. The Fourth Industrial Revolution (IR 4.0) refers to the current era of technological advancements marked by interconnectedness, automation, machine learning, and real-time data. Emerging from the digital revolution, it significantly impacts various sectors, including ART. Here, IR 4.0 introduces advanced techniques like genetic editing and AI-driven embryo selection, revolutionizing fertility treatments like ICSI. But at the same time the paradox of ICSI is that it kindles the issue of ‘andrological ignorance’ and may contribute to genetic defects in offspring.

Research indicates that there is a prevalent ‘andrological ignorance’ among clinicians who frequently opt for ICSI even in instances where it may not be necessary, primarily because of its perceived simplicity and efficiency [7–9]. The primary objective in fertility medicine is to address couples’ fertility challenges without exposing them to undue risks or ineffective treatments. For optimal medical practices, robust scientific evidence is indispensable. However, even with such evidence, it is not universally implemented, particularly in assisted reproduction. This field increasingly exhibits commercial tendencies, often diagnosing and treating beyond actual necessity [10]. ICSI, originally intended for poor sperm quality, is now frequently employed even when semen parameters are normal, becoming almost the default in IVF procedures. Research indicates that ICSI does not enhance live birth rates compared to conventional IVF when unrelated to male infertility [11]. Moreover, techniques like preimplantation genetic testing and specialized embryo incubation systems are used without substantial evidence of their benefits [12, 13].

## Main text

### Transcending ‘andrological ignorance’: role of AI in IR 4.0

In the era of IR 4.0, where AI and big data analytics are transforming healthcare, andrological knowledge lags other specialties. This is particularly evident in the field of male infertility, where the diagnosis and management of male factor infertility rely heavily on subjective assessments of sperm quality and quantity [14]. Advanced technologies, such as microfluidic sperm sorting, sperm DNA fragmentation analysis, and sperm proteomics, are emerging, but their implementation in clinical practice is limited by ‘andrological ignorance’ as well as insufficient trained andrologists critical for proper case-analysis and safe deployment of any emerging technology [15]. The lack of andrological knowledge and advanced technologies in male infertility diagnosis and management contributes to the ICSI paradox by perpetuating the reliance on ICSI as the preferred method of fertilization in severe male factor infertility cases [3]. Therefore, the issue of male infertility frequently does not get the necessary focus and consideration in terms of diagnosis and treatment. This often happens because medical professionals typically choose ICSI as a go-to solution, primarily due to its perceived simplicity and time efficiency. Such preference may be attributed to a lack of extensive understanding among clinicians about how to evaluate and address male infertility. Moreover, couples who are seeking solutions often lack awareness of alternatives to ARTs, limiting their potential options. This preference prevails despite the potential risks associated with ICSI, which include genetic aberrations, epigenetic modifications, and an elevated likelihood of imprinting disorders [2, 6].

Male infertility can arise from a multitude of factors such as genetic mutations, environmental toxins, and reversible causes like varicocele, infections, ejaculation issues, hormonal imbalances, tumors, undescended testicles, sperm transport defects, medication/substance use, and lifestyle factors [16]. Ignoring these potential causes may have a significant impact on the overall health and quality of life of the patient. Genetic mutations that cause male infertility may also predispose an individual to other health conditions. For example, mutations in the cystic fibrosis transmembrane conductance regulator (CFTR) gene that causes cystic fibrosis can also affect the male reproductive system, leading to infertility [17]. Additionally, mutations in genes associated with testosterone production or function can result in low levels of the hormone, which can lead to a variety of health issues such as decreased muscle mass and bone density, mood disorders, and increased risk of heart disease [18]. Sexually transmitted infections (STIs) can damage the male reproductive system, potentially causing infertility and other health implications, such as increased cancer risk and chronic diseases, if left untreated [19]. Research indicates a correlation between male infertility and heightened testicular cancer risk, especially in cases of severe infertility, possibly due to shared risk factors like exposure to environmental toxins [20]. Unaddressed male infertility often leads to psychological stress, such as anxiety, depression, and social isolation, negatively affecting mental health and overall quality of life. Therefore, investigating and managing potential causes of male infertility is crucial not only for improving fertility but also for overall men’s health [21].

AI and IR 4.0, which involve the integration of digital technologies and automation across various industries, offer promising solutions for addressing several challenges in male infertility diagnosis and treatment. Firstly, by analyzing medical records, semen analyses, and other diagnostic data, AI can identify patterns associated with male infertility, leading to more accurate diagnoses and personalized treatment plans [22]. AI can analyze complex data patterns linked to male infertility, which often eludes conventional diagnostic methods. By leveraging the advanced pattern recognition capabilities of AI, healthcare professionals can achieve more precise diagnoses. This enables the creation of tailored treatment strategies, significantly enhancing the effectiveness and efficiency of infertility treatments for men. Thus, knowledge of specific causatives and detailed diagnosis may reduce the reliance on invasive procedures like ICSI and address the root causes of male infertility. Secondly, AI can be utilized to analyze genetic information from both the sperm and egg, which can help identify potential genetic defects that could be inherited by offspring [23]. This comprehensive view of the genetic health of the embryo has the potential to mitigate the risks associated with ICSI. Thirdly, AI can be used to develop educational platforms and content that raise awareness about male reproductive health, addressing the issue of andrological ignorance. Interactive tools, personalized learning experiences, and targeted information can help bridge the knowledge gap, (a) to facilitate remote consultations with andrologists and other fertility specialists, making expert knowledge more accessible and reducing geographical barriers to care, (b) to analyze large datasets to identify patterns and correlations that can help optimize treatments and predict outcomes. This can lead to better treatment planning and reduced reliance on ICSI, and (c) to accelerate research on male infertility and potential treatments [24, 25]. By analyzing vast amounts of data, AI can help researchers identify new treatment targets, develop novel therapies, and better understand the underlying causes of male infertility [24].

### Supposition of the debate and future perspectives

The field of andrology, the medical specialty that deals with male health, particularly related to the male reproductive system and urological issues, has often been overshadowed by the over- and misuse of ARTs, may be owing to lack of adequate number of trained andrologists. This, however, has resulted in a gap in knowledge often referred to as ‘andrological ignorance’. It is crucial to acknowledge that couples undergoing fertility treatments, particularly those involving ARTs like ICSI, should receive comprehensive counseling about the potential risks and implications of these procedures. This counseling is essential to ensure informed decision-making and to prepare couples for all possible outcomes. The importance of such counseling underscores the need for a robust foundational knowledge in andrology, as it informs both the medical advice given and the decision-making process of patients. With the exponential rise of AI in healthcare, there is potential to rectify this issue and address male infertility more effectively. The strength of AI lies in its capacity to handle large volumes of complex data, to discern patterns, and to make predictions that might be beyond human perception. Its application in andrology could transform how clinicians and patients approach male fertility treatments and the usage of ARTs, particularly ICSI, which is often excessively used as the *de facto* solution to male infertility. However, a more targeted approach could improve its usage, ensuring that it is only used, when necessary, as ICSI carries potential risks and has a significant cost burden. Here, AI can be instrumental in enhancing the decision-making process. For instance, an AI system can be trained to predict the success rate of ICSI based on parameters such as sperm parameters, the age and health of the patient, history of previous fertility treatments, and so on. By taking into consideration a broad range of factors, the system could provide a comprehensive analysis and indicate when ICSI should be used, thereby optimizing its usage, and avoiding unnecessary procedures.

In addition to ICSI, AI can be used to prioritize male fertility treatments more broadly. There is ongoing research in AI-assisted semen analysis that leverages machine learning algorithms to predict male fertility. These technologies, such as the MOJO AI Sperm Analysis (MOJO AISA), can accurately and rapidly assess multiple sperm parameters, including count, motility, and morphology [26]. The conventional manual semen analysis is subjective and can have significant variability between observers. AI-assisted analysis, on the other hand, can potentially provide more consistent and reliable results [27]. Another example of potential of AI in the field of male infertility can be seen with the use of convolutional neural networks (CNNs), a type of AI, for automated sperm analysis [28]. These systems are trained on a large database of sperm images and can identify sperm based on their shape and movement patterns. They are also able to distinguish between normal and abnormal sperm, providing a more detailed analysis of sperm quality [28]. Such technologies can be used in tandem with other diagnostic tools, such as genetic testing, to provide a holistic view of a patient’s fertility status. This can then inform treatment decisions, ensuring that patients receive the most appropriate care for their specific circumstances.

The effective implementation of AI in andrology necessitates a sequential approach to build a robust knowledge base. Before integrating AI tools, it is imperative to have a comprehensive understanding of male reproductive health and the complexities of fertility treatments. This ensures that AI tools are developed and applied on a solid foundation of domain-specific knowledge, leading to more accurate and effective outcomes in patient care.

Further research is needed to validate the effectiveness of these AI technologies in clinical settings. But the preliminary work suggests that AI can play a pivotal role in overcoming andrological ignorance, improving the usage of ICSI, and prioritizing male fertility treatments. In the future, it is likely that AI will become an integral part of the clinical decision-making process in andrology and reproductive medicine. However, striking a balance between innovation and comprehensive care is crucial for optimizing patient outcomes in the rapidly evolving field of Reproductive Medicine. Addressing these issues will not only improve the outcomes of ART but also promote the long-term health and well-being of future generations.

## Data Availability

Not applicable.

## Abbreviations

AI: Artificial intelligence
ART: Assisted reproductive technology
CFTR: Cystic fibrosis transmembrane conductance regulator
CNN: Convolutional neural network
DNA: Deoxyribonucleic acid
ICSI: Intracytoplasmic sperm injection
IR: Industrial revolution
IVF: In vitro fertilization
MOJO AISA: MOJO artificial intelligence sperm analysis
